# Alcohol consumption during pregnancy and perinatal results: a cohort study: Consumo de álcool durante a gravidez e resultados perinatais: um estudo de coorte

**DOI:** 10.1590/1516-3180.2015.02040211

**Published:** 2016-03-15

**Authors:** Mariana Sbrana, Carlos Grandi, Murilo Brazan, Natacha Junquera, Marina Stevaux Nascimento, Marco Antonio Barbieri, Heloisa Bettiol, Viviane Cunha Cardoso

**Affiliations:** I MD. Medical Resident, Department of Pediatrics, Ribeirão Preto Medical School, Universidade de São Paulo (USP), Ribeirão Preto, São Paulo, Brazil.; II MD, PhD. Postdoctoral Student, Department of Gynecology and Obstetrics, Ribeirão Preto Medical School, Universidade de São Paulo (USP), Ribeirão Preto, São Paulo, Brazil.; III MD, PhD. Senior Professor, Department of Pediatrics, Ribeirão Preto Medical School, Universidade de São Paulo (USP), Ribeirão Preto, São Paulo, Brazil.; IV MD, PhD. Associate Professor, Department of Pediatrics, Ribeirão Preto Medical School, Universidade de São Paulo (USP), Ribeirão Preto, São Paulo, Brazil.; V MD, PhD. Adjunct Professor, Department of Pediatrics, Ribeirão Preto Medical School, Universidade de São Paulo (USP), Ribeirão Preto, São Paulo, Brazil.

**Keywords:** Alcohol drinking, Pregnancy, Infant, low birth weight, Infant, small for gestational age, Premature birth

## Abstract

**CONTEXT AND OBJECTIVE:**

Alcohol consumption during pregnancy is a significant social problem that may be associated with adverse perinatal outcomes. The aim of this study was to describe alcohol consumption during pregnancy and to study its association with low birth weight, newborns small for gestational age and preterm birth.

**DESIGN AND SETTING:**

Nested cohort study, in the city of Ribeirão Preto, São Paulo, Brazil.

**METHODS:**

1,370 women and their newborns were evaluated. A standardized questionnaire on health and lifestyle habits was applied to the mothers. Anthropometry was performed on the newborns. Alcohol consumption was defined as low, moderate or high, as defined by the World Health Organization. Adjusted logistic regression analysis was used.

**RESULTS:**

23% of the women consumed alcohol during pregnancy. Consumption mainly occurred in the first trimester (14.8%) and decreased as the pregnancy progressed. The median alcohol intake was 3.89 g (interquartile range, IQR = 8 g) per day. In the unadjusted analysis, alcohol consumption increased the risk of low birth weight almost twofold (odds ratio, OR 1.91; 95% confidence interval, CI: 1.25-2.92). The risk was lower in the adjusted analysis (OR 1.62; 95% CI: 1.03-2.54). Alcohol consumption did not show associations with small for gestational age or preterm birth. There was greater risk of low birth weight and newborns small for gestational age and preterm birth among mothers who were both smokers and drinkers.

**CONCLUSIONS:**

The alcohol consumption rate during pregnancy was 23% and was independently associated with low birth weight, but there was no risk of newborns small for gestational age or preterm birth.

## RESUMO

**CONTEXTO E OBJETIVO:**

O consumo de álcool durante a gravidez é um problema social significativo que pode estar associado a resultados perinatais adversos. O objetivo deste estudo foi descrever o consumo de álcool na gestação e avaliar sua associação com recém-nascido de baixo peso, pequeno para idade gestacional e pré-termo.

**TIPO DE ESTUDO E LOCAL:**

Estudo de coorte aninhado, na cidade de Ribeirão Preto, São Paulo, Brasil.

**MÉTODOS:**

Foram avaliadas 1.370 mulheres e seus recém-nascidos. Foi aplicado às mães um questionário padronizado sobre saúde e hábitos de vida. Antropometria foi realizada nos recém-nascidos. Consumo de álcool foi definido como baixo, moderado e elevado segundo a Organização Mundial de Saúde. Foi utilizada análise de regressão logística ajustada.

**RESULTADOS:**

23% das gestantes consumiram álcool durante a gravidez. A maior parte do consumo ocorreu no primeiro trimestre (14,8%) e diminuiu conforme progredia a gravidez. A mediana de ingestão de álcool foi de 3,89 g (interval interquartil, IIQ = 8 g) por dia. Na análise não ajustada, o consumo de álcool aumentou em quase duas vezes (*odds ratio*, OR 1,91, intervalo de confiança, IC 95%; 1,25-2,92) o risco de baixo peso, que se reduziu após ajuste (OR 1,62; IC 95%; 1,03-2,54). Não houve associação entre consumo de álcool e pequeno para idade gestacional ou pré-termo. Observou-se maior risco de baixo peso, neonato pequeno para idade gestacional e pré-termo em gestantes simultaneamente fumadoras e bebedoras.

**CONCLUSÕES:**

O consumo de álcool na gestação foi de 23% e esteve associado independentemente com o baixo peso ao nascer, mas não houve risco para neonato pequeno para idade gestacional e pré-termo.

## INTRODUCTION

Alcohol consumption during pregnancy is a significant social problem that may be associated with adverse perinatal outcomes such as low birth weight (LBW), small for gestational age (SGA) or preterm newborns.^1^ However, the relationship between alcohol consumption during pregnancy and adverse perinatal outcomes is still controversial. While some studies have detected higher percentages of LBW, SGA and preterm birth among infants exposed to alcohol during pregnancy,^2^^,^^3^^,^^4^^,^^5^ others have reported a reduced risk of SGA and preterm birth.^3^^,^^6^^,^^7^^,^^8^ In addition, consumption of large amounts of alcohol during pregnancy is associated with occurrence of fetal malformations,^9^ mental retardation^4^ and behavioral and psychosocial disorders during childhood and adolescence.^10^^,^^11^

LBW is a source of concern within healthcare because it is associated with higher neonatal and infant morbidity and mortality. Also, it is a heterogeneous condition because of two adverse conditions, i.e. prematurity and intrauterine growth restriction (IUGR), which may act to varying degrees either separately or synergistically.^12^^,^^13^

It has been reported that 20% to 65% of women consume alcohol at some time during pregnancy and that 5% to 10% consume levels sufficient to pose a risk to the fetus.^14^ There are also significant differences regarding the effects of maternal consumption of alcoholic drinks on fetal outcomes, according to the gestational period during exposure occurred, thus indicating the existence of critical periods, even for low levels of alcohol consumption.^3^ However, there is no difference in the level of risk among the different types of drinks (beer, wine and spirits).^15^

## OBJECTIVE

The objectives of the present study were to describe alcohol consumption during pregnancy and to assess its association with adverse perinatal outcomes (LBW, SGA and preterm birth) in a Brazilian birth cohort.

## METHODS

This nested cohort study formed part of a more extensive observational prospective study in which the main objective was to assess new risk factors for preterm birth and perinatal indicators and their impact on fetal and infant growth in two cohorts in the cities of Ribeirão Preto, São Paulo, and São Luís, Maranhão.^16^

The data used in the present study were from pregnant women who were evaluated during prenatal follow-up only in Ribeirão Preto and were reinterviewed at the time of their children’s births. Women were recruited at hospitals and healthcare units on the occasion of a prenatal visit during the first trimester of pregnancy, provided that an ultrasound examination performed up to the 20^th^ week of gestation was available in order to define gestational age. After being informed about the objectives of the study, the pregnant women were invited to the University Hospital of Ribeirão Preto Medical School, University of São Paulo (HCFMRP-USP), in order to participate in the study after giving their written informed consent. This evaluation was performed during the second trimester of pregnancy, at a gestational age of 20 to 25 weeks involving only singletons. A standardized questionnaire was applied to gather information on identification, reproductive health, maternal lifestyle habits such as alcohol consumption, and demographic and social characteristics. A standardized questionnaire was also applied to these women on the occasion of their childbirth, and anthropometric measurements on the newborn (weight, length and head circumference) were obtained through the medical records. Data gathering was started in January 2010 and ended in July 2011.

The primary outcomes were: i) LBW (birth weight < 2500 g); ii) relationship between birth weight and gestational age (GA), which was classified as SGA when the birth weight was below the 10^th^ percentile of the Williams curve of birth weight for GA;^17^ adequate for gestational age (AGA) when the birth weight was between the 10^th^ and 90^th^ percentiles; and large for gestational age (LGA) when the birth weight was above the 90^th^ percentile of the same curve; and iii) newborns with a GA of less than 37 completed weeks were classified as preterm.

The independent variable was alcohol consumption during pregnancy. The women were asked to report the frequency (as days per week), the amount (as number and type of glasses), the type of alcoholic drink consumed (beer, wine or spirits such as whisky, gin, vodka or rum) and the period of gestation during which alcohol intake occurred (first, second or third trimester). The amount of each drink consumed, in ml, was then calculated. This value was then converted to grams of absolute alcohol (alcohol density), taking into account the percentage of absolute alcohol present in each drink (5% absolute alcohol in beer, 12% in wine and 40% in spirits). When present, the maternal consumption of alcoholic beverages was classified as low (1 g to 20 g of absolute alcohol per day), moderate (21 g to 40 g of absolute alcohol per day) or high (41 g or more of absolute alcohol per day),^18^ and perinatal outcomes according to these levels of alcohol were calculated. For non-adjusted and adjusted analyses, alcohol consumption was transformed into a dichotomous variable (yes/no) because of the small number of moderate and high drinkers.^5^

The potential confounding variables included were maternal age (years), mother’s skin color (white or other), maternal schooling (completed years), marital status (with or without a partner), family head’s occupation (non-manual, skilled manual, semiskilled manual, unskilled manual or not within the economically active population),^19^ body mass index (BMI, kg/m^2^), maternal smoking during pregnancy and gestational hypertension.

**Figure 1.**
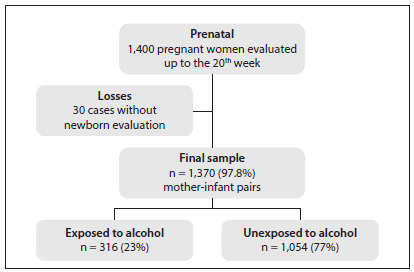
Flowchart of the study population, Ribeirão Preto, 2010.

The sample size was originally calculated by considering a 12% prematurity rate and a 10% error, with the significance level set at 0.05.^16^ A total of 1,400 women were included, but only 1,370 were assessed at two time points, i.e. in the second trimester of pregnancy and at delivery. However, using an estimate of alcohol consumption among Brazilian pregnant women of approximately 20%,^18^ with an odds ratio of 1.80, a type I error (α) of 0.05 and a type II error (β) of 0.20, 1,152 pregnant women would be necessary (768 unexposed and 384 exposed). A flow diagram of our study population is presented in Figure 1.

The descriptive statistics included the mean, median, proportion, standard deviation (SD), 95% confidence interval (CI) and interquartile range (IQR). Student’s t-test, analysis of variance (ANOVA) and the chi-square test were used to compare means and categorical variables.

Non-adjusted and adjusted logistic regression analyses were calculated to estimate the odds ratio (OR) and 95% CI, in order to express the associations of LBW, SGA and preterm birth with maternal alcohol consumption (yes/no, taking non-consumers as the reference). The data were controlled for potential confounders: maternal age, pregestational body mass index, maternal schooling, mother’s marital status, family head’s occupation, smoking during pregnancy and gestational hypertension. The goodness of fit of the logistic models was assessed by means of the Hosmer-Lemeshow test*.* In the next step, in order to assess unhealthy behavior, the effect of smoking on pregnancy was tested by means of an interaction term, with alcohol in another model. The adjusted population-attributable risk (aPAR), which assesses the proportion of cases that would not occur in a population if alcohol consumption were eliminated, was calculated using aPAR = 100 x (aOR - 1)/aOR + (100/ r) - 1, where *r* is the proportion of the population exposed to alcohol and aOR is the adjusted odds ratio.

Significance was set at P < 0.05 and all analyses were carried out using the Stata software, version 12.0 (College Station, Texas, USA).

The study was approved by the Research Ethics Committee of Hospital das Clínicas, Ribeirão Preto Medical School, University of São Paulo (Protocol n^o^ 11157/2008).

## RESULTS

Out of the 1370 women studied, 316 (23.0%; 95% confidence interval, CI: 20.9-25.3) consumed alcohol during pregnancy. Most of them had low intake, and beer was the most frequent type of beverage (Table 1).

**Table 1.**
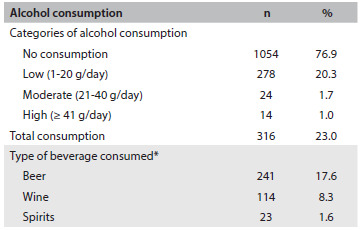
Alcohol consumption during pregnancy (Ribeirão Preto, 2010; n = 1,370)

*Some mothers consumed more than one type of beverage.

Only 5.1% of the pregnant women reported drinking throughout pregnancy. Alcohol consumption was 14.8% during the first trimester of pregnancy, with closely similar intake in the second and third trimesters (10.7% and 10.5%, respectively). Total alcohol consumption decreased with the progression of gestation, such that the mean intake was 57.1 ± 14.1 g in the first trimester, 41.6 ± 5.9 g in the second, and 35.9 ± 3.4 g in the third. The median daily alcohol intake during pregnancy among the women who reported drinking during pregnancy was 3.89 g (IQR 8 g).

Pregnant women who consumed spirits were the ones with the highest alcohol intake during pregnancy (mean of 40.7 ± 11 g), whereas those who consumed beer and wine had significantly lower alcohol intake (means of 7.7 ± 2.3 g and 5.3 ± 1.2 g, respectively) (P < 0.001).

A significant increase in alcohol consumption was observed with increasing maternal age. Women without a partner, smokers and those with hypertension during pregnancy also consumed alcohol in significant quantities. No difference was observed in relation to the other characteristics. between women who consumed alcohol and those who did not (Table 2).

**Table 2.**
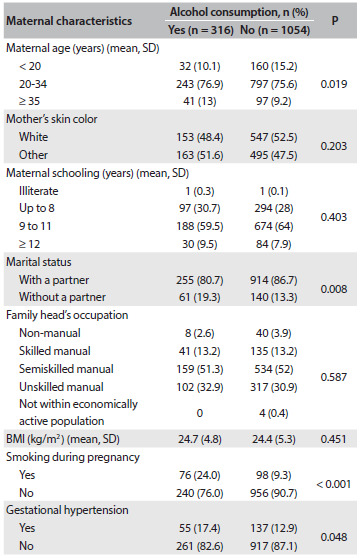
Comparison of maternal and gestational characteristics according to alcohol consumption (Ribeirão Preto, 2010)

BMI = body mass index.

The total prevalence of low birth weight was 7.6% (95% CI: 6.4-9.2), SGA 9.6% (8.2-11.3) and prematurity 9.7% (8.2-11.4). Among the LBW infants, 64.7% were preterm and 49.5% were SGA. Among the preterm infants, 11.2% were SGA.

Table 3 analyzes newborn characteristics according to alcohol consumption during pregnancy. The frequency of LBW almost doubled among mothers consuming alcohol (P = 0.002). On the other hand, there was no difference between babies born to mothers consuming alcohol and mothers who did not, in relation to mean gestational age, birth weight, SGA and preterm birth. Nevertheless, there was some dose-response relationship between alcohol consumption during pregnancy and the risk of low birth weight (6.4% for no consumption, 11.8% for low, 12.5% for moderate and 7.1% for high consumption; P = 0.020). No dose-response relationship was observed for SGA or preterm birth (P = 0.556 and 0.202, respectively).

**Table 3.**
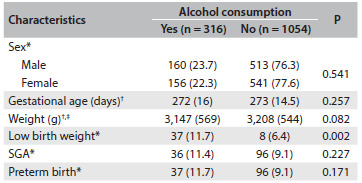
Characteristics of the newborns according to maternal alcohol consumption during pregnancy (Ribeirão Preto, 2010)

*n (%); ^†^mean (standard deviation); ^‡^three cases with no information about birth weight; SGA = small for gestational age.

Unadjusted analysis revealed that alcohol consumption during pregnancy increased the risk of LBW almost twofold (OR 1.91; 95% CI: 1.25-2.92), compared with its absence. After adjustment for confounders, the only perinatal outcome independently associated with alcohol consumption was LBW, although the OR was reduced to 1.62 (P = 0.034) (Table 4). The goodness of fit of the models was satisfactory, and the Hosmer-Lemeshow tests were nonsignificant (LBW = 0.894, SGA = 0.646 and prematurity = 0.656).

**Table 4.**
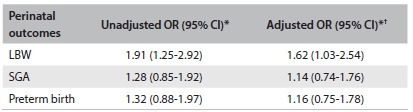
Unadjusted and adjusted risks according to sociodemographic characteristics and clinical conditions for different perinatal outcomes associated with alcohol consumption during pregnancy (Ribeirão Preto, 2010)

*reference: did not consume alcohol; ^†^adjusted for maternal age, pregestational BMI, maternal schooling, marital status of the mother, occupation of the family head, smoking during pregnancy and gestational hypertension; OR = odds ratio; LBW = low birth weight; SGA = small for gestational age.

The effects of smoking and alcohol consumption during pregnancy on neonatal outcomes were explored through interaction, and this revealed high adjusted risks of LBW (OR 3.65; 95% CI: 1.84-7.24; P < 0.001), SGA (2.56; 1.33-4.92; P = 0.005) and preterm birth (2.57; 1.35-4.89; P = 0.004) among women who simultaneously smoked and consumed alcohol, compared with those who did not smoke or drink.

The adjusted population-attributable risk of alcohol consumption was 12.5% for LBW, 3.1% for SGA and 3.5% for preterm birth.

## DISCUSSION

In the present study, 23% of the pregnant women consumed alcohol during pregnancy, most of them in a mild manner. A significant almost twofold increase in the risk of LBW was detected among these women, compared with those who never drank.

Internationally, the prevalence of alcohol consumption during pregnancy ranges from 30 to 70%.^20^^,^^21^ A study in Rio de Janeiro^22^ detected prevalence of 7.3% to 26.1%, depending on the screening method adopted, compared with 23% in the present study.

The relationship between alcohol consumption during pregnancy and adverse perinatal outcomes is a matter of controversy. A study conducted in Rio de Janeiro detected no relationship between alcohol consumption and preterm birth, LBW or negative effects on the newborn. However, this lack of association may have been due to the small sample size and to the limited information obtained about dose and frequency of consumption.^23^

A systematic review of low or moderate alcohol consumption did not showed any significant effects on the pregnancy outcomes considered (abortion, stillbirth, fetal growth restriction, preterm, SGA or malformations, including fetal alcoholic syndrome), although it should be pointed out that many of the studies reviewed had methodological deficiencies.^8^

In another systematic review, light to moderate alcohol consumption during pregnancy was not associated with preterm birth or SGA risks.^4^

About 12% of low birth weight cases may be attributed to alcohol consumption, but the aPAR of the present study was lower for SGA and PT and even lower than the levels reported by O’Leary et al.,^2^ considering that the latter included frequent drinkers and women who consumed alcohol up to the first trimester of pregnancy. Consequently, prevention of alcohol consumption before the beginning of pregnancy could minimize adverse perinatal outcomes and reduce healthcare budgets.

There are several possible explanations for the failure to detect an association between alcohol and occurrences of SGA and preterm birth. Although maternal characteristics and medical complications were included in the adjusted model, there may have been specific health conditions predisposing towards SGA or preterm birth that we were unable to investigate in the present study (e.g. previous spontaneous preterm labor, placental insufficiency, intrauterine infection and interactions between genetic and environmental factors). Another possible explanation could be the inhibitory effect of alcohol on uterine contractions: this reduces the release of vasopressin and oxytocin during labor^24^ and consequently delays the onset of labor.

Henderson et al.^8^ suggested that low amounts of alcohol seem to have a small protective effect on birth weight, but no effect on or any reduction of the risk of prematurity, with consumption of up to 72 g of alcohol per week. The authors proposed that one possible explanation for this finding might be a “healthy drinker effect”, whereby women with a poorer obstetric history or prognosis are more likely to abstain from alcohol consumption during pregnancy.

The physiopathology of how alcohol may induce low fetal weight may be related to prostaglandins, which play an important role regarding fetal development and birth. Animal models have demonstrated that alcohol increases the production of prostaglandins, including those of the E series. Prostaglandins increase cAMP activity, thus reducing cell division, and an association between high cAMP levels and LBW has been reported. In human beings, increased secretion of prostaglandins and thromboxanes has been detected among alcoholics and their descendants.^6^

In addition, the effects of alcohol consumption depend on its absorption and on mother and fetus metabolism,^25^ a circumstance that may be partly genetically determined. Finally, differences may also have been due to the polymorphisms linked to alcohol metabolism, which may vary between populations.^15^ Thus, the effects of alcohol consumption need to be further studied in specific subgroups of women.

Alcoholism during pregnancy may be underdiagnosed due to “feelings of guilt” among women who, in order to avoid possible disapproval and reprimands from society and from healthcare professionals, may report lower consumption or deny it, i.e. they may exhibit recall bias.^26^ Furthermore, it is possible that women who report ceasing to drink during pregnancy actually did not do so. In the present study, this bias was minimized by using questionnaires applied at two different times (during pregnancy and postnatally). This method is used in order to improve the validity^27^ and accuracy^28^ of determinations of alcohol exposure.

There are various methods for assessing alcohol consumption, such as analysis of biomarkers in blood, direct interviews and filling out questionnaires. Since these biomarkers and ethanol metabolites are removed relatively rapidly from the bloodstream, they are poor indicators of exposure to alcohol, especially in populations that have low to moderate intake of this substance, as was the case in the present study. Thus, data obtained through interview are the most reliable way to assess mild and moderate levels of alcoholic beverage consumption.^3^

Another difficulty is that alcohol consumption is typically measured through the mean number of drinks per week or month. However, this may mask exposure to peak blood levels of alcohol (caused by binge drinking) and the exact timing of the exposure.^29^ In the present population, mild consumption was the most frequent finding (87.9% of the women who drank), but the potential risk to the fetus needs greater depth of investigation. The possible explanations for the lack of concordance between the findings from the various studies include low statistical power to detect small effects and inappropriate characterization of patterns and timing of alcohol consumption.

Smoking, which is an indicator of unhealthy behavior, has been reported to be a modifier of the effect of alcohol consumption on infant birth weight.^30^^,^^31^ The present results showed significant interaction between alcohol consumption and smoking with regard to the risk of LBW, SGA and preterm birth. Smoking and stress may create a biological environment in which alcohol has more adverse effects due to interactive mechanisms. Several biochemical interactions caused by alcohol and tobacco, with vitamins, folates and other antioxidants, may affect fetal growth during pregnancy.^3^^,^^31^ In addition, the vasoconstriction of the placenta-umbilical cord unit due to alcohol and tobacco consumption reduces the rate of alcohol elimination from the fetal compartment.^25^

Several limitations of the present study need to be mentioned. Firstly, it should be noted that the sample size was originally not computed for alcohol consumption,^16^ and therefore a type 1 error cannot be ruled out. However, we think that the minimal differences between the estimated and observed sample size would not modify the results. Secondly, alcohol intake during pregnancy was measured on a self-reported basis, which is known to underestimate the frequency and quantity of alcohol consumed by pregnant women. Thirdly, selective participation may have occurred, given that the women who consumed alcohol during pregnancy were in a less healthy condition (Table 2) and probably had a higher frequency of non-response regarding high alcohol intake. This may have contributed towards the lack of evidence of any adverse effect from alcohol intake on SGA or preterm birth.^32^^,^^33^^,^^34^ Finally, because the women were asked about their alcohol intake at 20-25 weeks of pregnancy, some recall bias regarding alcohol intake during the first trimester of pregnancy is possible.

The strength of the present study was that it assessed a birth cohort that had been followed up since the prenatal period, with a high response rate. Since data were collected at two time points, we were able to confirm the information and to adjust it for a wide range of known confounders, such as maternal behavior and sociodemographic factors. Another contribution was that smoking during pregnancy was included in this study. This is a major confounding factor regarding the relationship between maternal alcohol consumption and adverse perinatal outcomes.

## CONCLUSIONS

The results reported here indicate that alcohol consumption gave rise to increased risk of low birth weight, but no risk of SGA or preterm birth among the infants born to these mothers. We observed higher risk of low birth weight, SGA and preterm birth among infants born to mothers who smoked in addition to consuming alcohol during pregnancy. The combined effect of smoking and alcohol needs to be taken into account when counseling women about healthy behavior before and during pregnancy.
